# Unveiling the impact of cryptic plasmids curing on *Escherichia coli* Nissle 1917: massive increase in Ag43c expression

**DOI:** 10.1186/s13568-024-01681-9

**Published:** 2024-04-28

**Authors:** Qi Lin, Zhuo Jiang, Bo Zhong, Jian-qing Chen, Zheng-bing Lv, Zuo-ming Nie

**Affiliations:** 1 College of Life Sciences and Medicine, Zhejiang Sci-Tech University, Hangzhou, 310018 China; 2 Center for Bioreactor and Protein Drug Research, Shaoxing Biomedical Research Institute of Zhejiang Sci-Tech University Co., Ltd, Shaoxing, 312075 China

**Keywords:** *Escherichia coli* Nissle 1917, Cryptic plasmids, Transcriptome, Ag43

## Abstract

*Escherichia coli* Nissle 1917 (EcN) is an important chassis strain widely used for the development of live biotherapeutic products (LBPs). EcN strain naturally harbors two cryptic plasmids with unknown function. During the development of LBPs using EcN strain, the cryptic plasmids were cured usually to avoid plasmid incompatibility or alleviate metabolic burdens associated with these cryptic plasmids. While the cryptic plasmids curing in EcN may appear to be a routine procedure, the comprehensive impact of cryptic plasmids curing on the EcN strain remains incompletely understood. In the present study, the effects of cryptic plasmids curing on EcN were investigated using transcriptome sequencing. The results revealed that only a small number of genes showed significant changes in mRNA levels after cryptic plasmid curing (4 upregulated and 6 downregulated genes), primarily involved in amino acid metabolism. Furthermore, the *flu* gene showed the most significant different expression, encoding Antigen 43 (Ag43) protein, a Cah family adhesin. Mass spectrometry analysis further confirmed the significant increase in Ag43 expression. Ag43 is commonly present in *Escherichia coli* and mediates the bacterial autoaggregation. However, despite the upregulation of Ag43 expression, no Ag43-mediated cell self-sedimentation was observed in the cured EcN strain. These findings contribute to making informed decisions regarding the curing of the cryptic plasmids when *Escherichia coli* Nissle 1917 is used as the chassis strain.

**Supplementary Information:**

The online version contains supplementary material available at 10.1186/s13568-024-01681-9.

## Key points

- The PCR method was used to identify the EcN mutant strain (EcNc) of cured cryptic plasmids.
- Compared to the EcN strain, a significant upregulation in the expression of Ag43 was observed in the EcNc strain.
- The Ag43 protein up-regulated in the EcNc strain is a variant that does not mediate autoaggregation of bacterial cells.

**Supplementary Information:**

The online version contains supplementary material available at 10.1186/s13568-024-01681-9.

## Introduction

*Escherichia coli* Nissle 1917 (EcN), originally isolated by Alfred Nissle in 1917, has been used as the active ingredient in Mutaflor®, a pharmaceutical preparation used in the treatment of diverse diseases and intestinal dysfunctions (Sonnenborn and Schulze 2009). Due to its extensive history in medicinal use and the genetic tractability inherent in *E. coli*, EcN has emerged as a primary chassis strain. Researchers have undertaken modifications to enhance the functional capabilities of the EcN strain (Dong et al. 2023). Noteworthy outcomes of EcN modifications include the expression of the SARS-CoV-2 spike protein (SP) in EcN to immunize mice and induce antibody production (Sarnelli et al. 2023), as well as in situ expression of enzymes within the phenylalanine degradation pathway in the intestine for the treatment of phenylketonuria (Adolfsen et al. 2021). Additionally, heterologous expression of immunomodulatory factors in EcN has been effective for the treatment of ulcerative colitis. These factors include trefoil factors (TFFs) (Praveschotinunt et al. 2019), catalase and superoxide dismutase (Zhou et al. 2022), and Elafin (Teng et al. 2022).

EcN harbors two stable cryptic plasmids, denoted as pMUT1 and pMUT2, with the length of 3.2 Kb and 5.5 Kb, respectively (Sonnenborn and Schulze 2009). pMUT1 has a typical ColE1 replication origin, while pMUT2 has a cole2-like replication site and a ColE1-like mobilization site (Avison et al. 2001; Blum-Oehler et al. 2003). There are 6 and 11 potential ORFs on pMUT1 and pMUT2 plasmids, respectively. Both plasmids contain *Mob* genes associated with plasmid transfer. The pMUT2 plasmid also contains the *relB-relE* toxin-antitoxin system, commonly found in plasmids, playing a role in maintaining plasmid stability. In addition to these genes, the other putative genes in both plasmids mostly have no known functions (Kan et al. 2020). In LB culture medium at 37 °C, the copy number of pMUT1 was approximately 132 ± 11, while that of pMUT2 was about 97 ± 36.3 (Zainuddin et al. 2019). Despite the presence of these cryptic plasmids, their specific roles within the EcN context remain elusive and do not manifest any observable phenotypic alterations in EcN (Kan et al. 2020).

Some researchers have speculated that the presence of cryptic plasmids may impose a metabolic burden. Alternatively, others have considered the necessity of eliminating cryptic plasmids to avoid plasmid incompatibility when introducing a new plasmid. As a result, they chose to cure the cryptic plasmids (Zainuddin et al. 2019). There are two methods to cure cryptic plasmids based on plasmid incompatibility and CRISPR-cas9 described in previous reports (Kan et al. 2020; Liu et al. 2012; Zainuddin et al. 2019). The EcN mutant strain, resulting from the curing of both cryptic plasmids, is called as EcNc (Zainuddin et al. 2019).

In previous studies, comparative analyses between EcN and EcNc were conducted, focusing on morphology, growth, and metabolism. The outcomes revealed no observable differences between the two strains in these aspects (Sonnenborn and Schulze 2009). A subsequent study showed that EcN and EcNc had similar intestinal colonization in mice (Remer et al. 2009). Another study explored antibiotic production in both EcN and EcNc, yielding qualitatively similar results (Seo et al. 2012). In recent years, the potential impact of cryptic plasmids on heterologous protein expression in EcN was explored. It was observed that the presence of the native plasmid altered gene expression from the engineered plasmid significantly (Kan et al. 2020).

While the cryptic plasmids curing in EcN may appear to be a routine procedure, the comprehensive evaluation of its impact on the EcN strain has been lacking. The two cryptic plasmids have demonstrated stability in the absence of antibiotic pressure, leading us to hypothesize that these cryptic plasmids in EcN serve a biological function. Therefore, it is necessary to investigate the effects of cryptic plasmids curing on EcN to gain a more thorough understanding. In this study, we initially cured the cryptic plasmids using the principle of plasmid incompatibility. Subsequently, three pairs of primers were designed to identify the EcNc strain. Upon obtaining both EcN and EcNc strains, transcriptome sequencing was conducted. Through transcriptome analysis, discernible differences were observed in the transcriptional profiles of genes between the EcN and EcNc strains, subsequently validated through qPCR. Following this, differential analysis was extended to the protein level, and an initial exploration of phenotypic distinctions was also undertaken. These findings contributed to the understanding of the function of the two cryptic plasmids in *Escherichia coli* Nissle 1917.

## Materials and methods

### Strains and plasmids

The EcN strain (DSMZ: Cat# DSM6601) was purchased from BIOBW (BIOBW, Beijing, China). The *E. coli* BL21 and DH5α strains were originated from GENEWIZ (GENEWIZ, Suzhou, China). The pRE112 suicide plasmid with chloramphenicol resistance gene was purchased from Fenghui Biology (Fenghui Biology, Changsha, China).

### Construction of recombinant suicide plasmid by in-fusion cloning

The linear pMUT1 and pMUT2 were amplified by inverse PCR using pMUT1 and pMUT2 plasmids in wild EcN as a template. The linear pRE112 with homologous arm was amplified by inverse PCR using pRE112 plasmid in SM10λpir strain as a template. The recombinant suicide plasmids, pRE112-pM1, and pRE112-pM2, were constructed by the One Step Cloning Kit (Yeasen Biotech, Shanghai, China). The above used primers were listed in Additional file 2: Table S1.

### Curing of endogenous cryptic plasmids by plasmid incompatibility

The cryptic plasmids in EcN were cured through plasmid incompatibility (Liu et al. 2012). Briefly, the above constructed pRE112-pM1 was introduced into EcN competent cells by electroporation method. Under the pressure of chloramphenicol, pMUT1 was cured by plasmid incompatibility. Then the EcN strains containing pRE112-pM1 were induced to commit suicide by adding 10% sucrose to LB medium to obtain a mutant EcN strain containing only pMUT2 plasmid. The same method was used to cure pMUT2 to obtain EcNc strain. PCR was performed using two pairs of primers (muta5/6-F, muta5/6-R, muta7/8-F, muta7/8-R) that specifically bind to the cryptic plasmids to confirm that the cryptic plasmids have been cured. The used primers were listed in Additional file 2: Table S1.

### Design of three pairs of primers to specifically identify the genome of EcN strain

By comparing the gene annotations of the EcN genome (GCF_021559835.1) with that of the commonly used *E. coli* strain, BL21 (GCF_014263375.1) and DH5α (GCF_000982435.1), the unique genes exclusive to EcN were obtained. Subsequently, these unique genes underwent a thorough verification of their distinctiveness by conducting a BLAST search against the *E. coli* database. Among the unique genes, two genes exhibiting minimal homologous sequences were selected for primer design respectively. Then a pair of primers were designed according to the conserved housekeeping gene *gapA* in *E. coli*. These three pairs of primers have similar Tm values and similar amplification product sizes, which can be used for multiplex PCR.

### Transcriptome data analysis

The EcN and EcNc strains were streaked on LB agar plates without antibiotics to obtain isolated single colonies. These colonies were then inoculated into LB liquid medium and cultured on a shaker at 37 °C, 220 rpm until reaching the logarithmic growth phase. The cells were centrifuged and sent to Shenzhen BGI genomics for transcriptome sequencing.

Following the acquisition of raw data, RNA-seq reads were processed using the hisat2 (2.2.1) and htseq (2.0.2) tools, aligning them to the latest EcN reference genome (GCF_021559835.1) (Kim et al. 2019; Putri et al. 2022). Differential expression analysis was performed using DESeq2 (1.40.2) (Love et al. 2014). Criteria for identifying differentially expressed genes included a log2 (fold change) absolute value ≥ 1.0 and a multiple hypothesis test-corrected *p*-value ≤ 0.05. GO analysis about differentially expressed genes was performed using clusterProfiler (4.8.2) (Wu et al. 2021). These sequence data have been submitted to the Sequence Read Archive (SRA) databases under Bioproject accession PRJNA1045399.

### qPCR validation

The two strains (EcN and EcNc) were inoculated into LB medium at a 1% inoculation rate, and the cultures were agitated on a shaker until reaching the logarithmic growth phase before harvesting. Total RNA extraction was carried out using a total RNA extraction kit (Genstone Biotech, Beijing, China) according to the manufacturer’s protocol. Reverse transcription was conducted using a reverse transcription kit (Accurate Biotech, Changsha, China), and qPCR validation was performed using a qPCR kit (Yeasen Biotech, Shanghai, China) following the manufacturer’s protocol. The 2^ΔΔCt^ method was employed for data analysis. The qPCR primers employed can be found in Additional file 2: Table S2.

### Identification of differentially expressed protein by mass spectrometry

The two strains of EcN and EcNc were cultured to the logarithmic growth phase and harvested. Bacterial pellets were collected by centrifugation at 12,000 rpm for 3 min, washed with PBS, resuspended, and heated at 60 °C for 30 min. The supernatant obtained after centrifugation was considered as heat-extracted protein. Following SDS-PAGE and Coomassie blue staining, the differential protein band was excised and sent to Shanghai Luming Biotechnology Co., Ltd. for mass spectrometry identification. Initially, enzymatic digestion was applied to protein band, followed by a C18 desalting treatment. Subsequently, the processed sample was subjected to identification using a liquid chromatography-tandem mass spectrometry system including the Ultimate 3000nano ultra-high-performance liquid chromatography and Q Exactive Plus high-resolution mass spectrometer (Thermo, Massachusetts, USA). The acquired raw data from mass spectrometry analysis were processed and analyzed using Thermo Proteome Discoverer software (Thermo, Massachusetts, USA). The target protein database from NCBI (https://www.ncbi.nlm.nih.gov/nuccore/NZ_CP022686.1, Proteins from EcN genome) was employed for retrieval, and the search parameters were set as follows: trypsin digestion, a mass tolerance of 10 ppm for the first-level mass spectrometry, and 0.05 Da for the second-level mass spectrometry. The peptide false discovery rate (FDR) is set at < 0.01, and the protein FDR is set at < 0.05. Under these criteria, a protein is considered to be present if it is mapped with at least one unique peptide which exhibits associated tandem mass spectrometry (MS/MS) spectra. For the mass spectrometry identification, two independent repeats were performed (Additional file 2: Tables S4 and S5).

### Aggregation test

According to the description in the report, aggregation assays were conducted for the EcN and EcNc strains (Hasman et al. 1999). Briefly, the two strains of EcN and EcNc were cultured to the logarithmic growth phase at 37 °C, 220 rpm. The bacterial cultures were adjusted to approximately uniform optical density at 600 nm (OD_600_). Subsequently, 5 mL of the bacterial culture was transferred into sterile 15 mL centrifuge tubes. After brief vortexing, the cultures were allowed to stand at room temperature. At 30-min intervals within a 120-min period, 200 µL samples were extracted from the uppermost layer of the bacterial culture, and their optical density at OD_600_ was determined.

### Statistical analysis

Statistical analyses and graphical representations were performed using GraphPad Prism software (Version 8.0). Group differences were assessed through t-tests (n = 3). Statistical significance was denoted as *p < 0.05, **p < 0.01, and ***p < 0.001, indicating a difference, significant difference, and extremely significant difference, respectively. Row means with SEM.

## Results

### Curing of the cryptic plasmids by using the incompatible nature of the plasmid

**Fig. 1:**
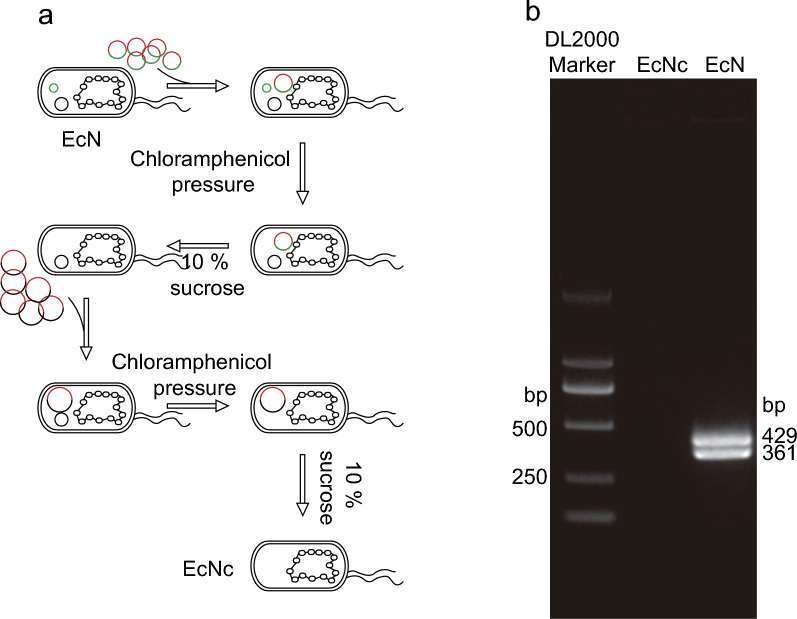
Cryptic plasmid curing. **a** The schematic diagram of the cryptic plasmid curing process. **b** The curing of the cryptic plasmids was identified by PCR. PCR was performed using two pairs of primers (muta5/6-F, muta5/6-R, muta7/8-F, muta7/8-R) that were listed in Additional file 2: Table S1

To investigate the impact of the curing of cryptic plasmids on the EcN strain, we explored the differences at the transcriptional expression level. To obtain an EcN strain with the cryptic plasmids curing, we constructed two recombinant suicide plasmids which contained the same origin of replication (ori) with the cryptic plamids. These plasmids were sequentially transformed into EcN to cure the cryptic plasmids based on plasmid incompatibility, and then the sucrose was added to trigger their self-destruction (Fig. 1a). The recombinant suicide plasmids contains the *sacB* gene encoding levansucrase. When the EcN strain harboring the recombinant suicide plasmids is cultivated on plates containing 10% sucrose, levan can be produced by the hydrolysis of sucrose through levansucrase, and accumulates in the periplasmic space of EcN, hindering bacterial growth, thus EcNc strains that do not harbor any plasmids could be screened (Ying et al. 2016). Subsequently, the curing of cryptic plasmids was confirmed using PCR with specific primers designed based on previous studies (Blum-Oehler et al. 2003). The results showed bands of 429 and 361 bp in size for EcN, while absent in EcNc, indicating the successful curing of pMUT1 and pMUT2 in EcNc strain (Fig. 1b).

### Identification of EcNc strain

At present, the identification of the EcN strain is commonly performed using three pairs of specific primers that target the combination of two cryptic plasmids (Blum-Oehler et al. 2003). However, these three primer sets fail to identify EcNc strains. Additionally, the identification based on 16S rRNA can only provide basic identification at the species level. The EcNc strain is a non-antibiotic resistant strain of *E. coli*. Therefore, there is a need for a more convenient, rapid, and accurate identification method to identify EcNc.

**Fig. 2:**
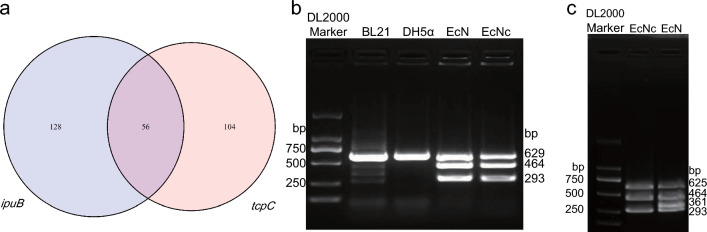
Identification of primer design for EcN genome. **a** Venn diagram of blast results for *ipuB* and *tcpC*. The blast results for *ipuB* revealed similarities in only 184 strains, while the blast results for *tcpC* showed similarities in only 160 strains (as of September 9, 2023). Of these, 56 strains were found to overlap between the 184 strains with *ipuB* similarity and the 160 strains with *tcpC* similarity. **b** Primer verification. PCR amplification was performed on the BL21, DH5α, EcN, and EcNc strains using three pairs of designed primers. **c** Identification of EcN and EcNc strains. Incorporating a pair of primers (muta5/6-F and mata5/6-R) designed for identifying cryptic plasmids into the three primer pairs, PCR amplification was performed to further distinguish between EcN and EcNc

Three pairs of primers were designed based on the EcN genome for the identification of EcN and EcNc strains through multiplex PCR. Initially, 115 unique genes were identified in EcN, which not existed in the BL21 and DH5α genomes. Subsequently, two genes, *ipuB* and *tcpC*, were screened from these 115 genes. The product of *ipuB* is tyrosine-type DNA invertase, while the product of *tcpC* is NAD (+) hydrolase. These two genes were selected because the number of *E. coli* strains harboring both them is the smallest (only 56, Fig. 2a, Additional file 2: Table S3). Consequently, primers were designed for the genes, *ipuB* and *tcpC*, and the length of PCR products was 293 bp and 464 bp, respectively. Additionally, a pair of primers were designed for the housekeeping gene *gapA* in *E. coli*, producing a PCR product of 629 bp. The results of PCR revealed that the bands were appeared at 293 bp, 464 bp, and 629 bp for both EcN and EcNc, whereas only the 629 bp band of the housekeeping gene was exhibited for BL21 and DH5α (Fig. 2b). This suggested that the designed primers can be used for the specific identification of EcN and EcNc strains. To further differentiate between EcN and EcNc, one pair of primers targeting a cryptic plasmid was selected (muta5/6-F and mata5/6-R). The PCR amplification exhibited a 361 bp band of the cryptic plasmid for EcN, and no band exhibited for EcNc strain (Fig. 2c), showing the ability to distinguish between EcN and EcNc.

### Transcriptome analysis and qPCR verification of EcN and EcNc

**Fig. 3:**
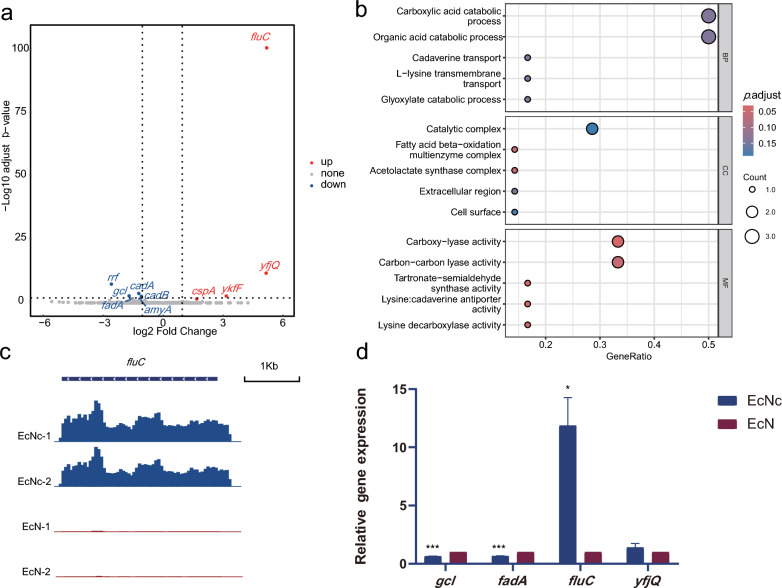
Transcriptome data analysis and qPCR verification of EcN and EcNc. **a** Differential expressed gene volcano map on the genome. **b** GO enrichment analysis of differential expressed genes on the genome. *BP* biological process, *CC* cellular component, *MF* molecular function. **c** Statistical visualization of *fluC* reads. The reads were normalized as Fragments Per Kilobase per Million (FPKM). **d** Four genes were verified by qPCR. *Gcl*, *fadA*, *fluC*, and *yfjQ* encoded glyoxylate carboligase, 3-ketoacyl-CoA thiolase, self recognizing antigen 43 (Ag43) autotransporter, and DUF932 domain-containing protein, respectively

Comparison of the transcriptome data between EcN and EcNc revealed some impact of cryptic plasmids curing on the genome transcription, affecting a small subset of genes—10 in total, with 6 downregulated and 4 upregulated (Fig. 3a). Gene Ontology (GO) enrichment analysis of these differentially expressed genes showed a predominant enrichment in the molecular function, mainly in amino acid metabolism, such as carboxy-lyase activity, carbon–carbon lyase activity, tartronate-semialdehyde synthase activity, lysine:cadaverine antiporter activity, and lysine decarboxylase activity (Fig. 3b).

The gene (*flu*/*agn43*) encoding the Ag43/Cah family adhesin exhibited the most significant differential expression (Fig. 3c). Ag43 is widely distributed in *E. coli* strains and typically encoded by multiple gene copies within a single strain (Roche et al. 2001). The EcN genome harbors three copies of the Ag43 gene (named *fluA*, *fluB*, *fluC*), expressing three Ag43 variants (named Ag43a, Ag43b, Ag43c), with the lengths of 1040 aa, 1039 aa, and 948 aa, respectively. Recent research has classified Ag43 variants into six distinct categories according to phylogenetic analyses, denoted as C1, C2, C3, C4, C5, and C6 (Ageorges et al. 2023). In EcN, Ag43a and Ag43b fall into the C3 category, while Ag43c belongs to the C6 category. Notably, after the cryptic plasmids curing, the expression level of the C6 category Ag43c significantly increased at the transcriptional level, while there was no apparent difference in the expression of the other two C3 category Ag43 genes, Ag43a, Ag43b.

Subsequently, we further validated the transcriptome data using qPCR, selecting four genes from the differentially expressed gene. Among these, two were significantly upregulated (*fluC* and *yfjQ*), and two were significantly downregulated (*fadA* and *gcl*). The *gapA* gene was used as an internal reference. The qPCR results exhibited a consistent trend with the transcriptome results (Fig. 3d), providing further confirmation that the cryptic plasmids curing led to a significant increase in the transcriptional expression of Ag43c.

### Massive increase in protein level of Ag43c after cryptic plasmids curing

**Fig. 4:**
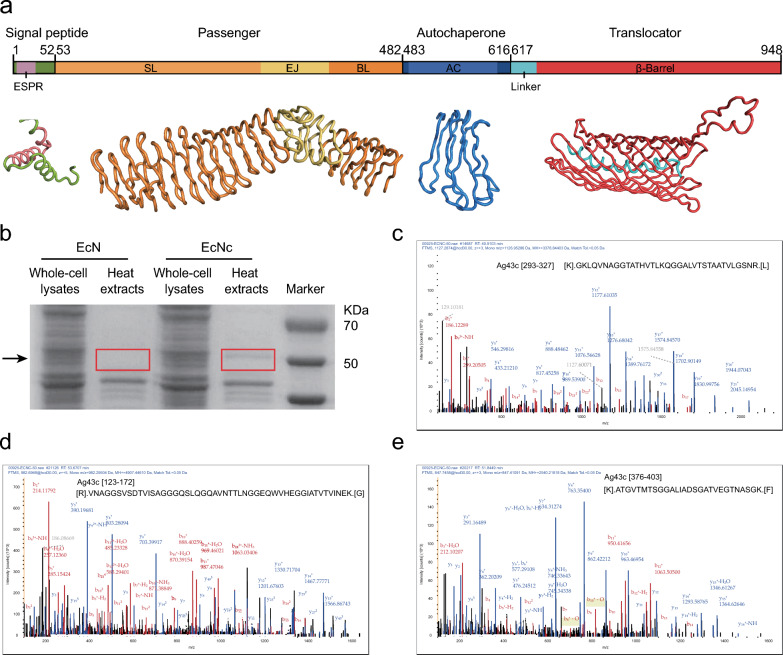
The differential level of Ag43 protein and mass spectrometry analysis. **a** Ag43c structure diagram. Ag43 features a modular structure, including: (1) the N-terminal signal sequence (SP), encompassing a highly conserved extension region (ESPR); (2) the passenger (α domain) responsible for protein function, which can be divided into three subdomains: SL (stem of the L shape), EJ (elbow joining), and BL (bottom of the L shape); (3) the autochaperone domain (AC); (4) the C-terminal β domain. The Ag43c structure was predicted by Robetta (https://robetta.bakerlab.org/). **b** The analysis of whole bacteria or heat-extracted proteins of EcN and EcNc by SDS-PAGE. **c**–**e** MS/MS spectra of peptides in Ag43c

The structure of Ag43c was predicted using Robetta (https://robetta.bakerlab.org/). Ag43c, like other variants of Ag43, possesses a modular structure (Fig. 4a), including: the N-terminal signal sequence (SP), the L-type passenger domain (α domain), the autochaperone domain (AC), the C-terminal translocator (β domain) (Ageorges et al. 2023; Benz and Schmidt 2011; Koh et al. 2022). Upon crossing the inner membrane, the SP sequence is cleaved (Ageorges et al. 2019; Koh et al. 2022). After traversing the outer membrane, the region between the AC and β domain is cleaved by an unknown protease (Ageorges et al. 2023). The Ag43α domain attaches to the cell surface through non-covalent interactions and can be easily released through simple heat treatment (Henderson et al. 2004; Klemm et al. 2003; Owen et al. 1996).

The cultures of EcN and EcNc were incubated at 60 °C for 30 min, followed by centrifugation. The supernatant was collected as the heat-extracted proteins and was detected by SDS-PAGE. Interestingly, by comparing the heat-extracted proteins from the EcN and EcNc strains, a distinct band in the range of 50 kDa to 70 kDa is observed, consistent with the anticipated range of 54.4 kDa to 60.1 kDa of Ag43c (Fig. 4b). Subsequent mass spectrometry analysis for this distinct band showed the presence of three proteins, Ag43a, Ag43b, and Ag43c (Fig. 4c–e, Additional file 1: Fig. S1). However, the relative abundance of Ag43c was 462.9 times that of Ag43a and 2740.7 times that of Ag43b. Furthermore, the relative abundance of Ag43c was at least 9.5 times higher than that of other proteins (Additional file 2: Table S4). Thus, we concluded that the predominant protein within this distinct band is Ag43c, indicating that the elevated content of Ag43c is responsible for the observed differential band. Additionally, two recombinant cryptic plasmids expressing *SOD* gene were constructed, and the EcN mutant strain containing only the recombinant cryptic plasmids exhibited no differential band caused by Ag43c, similar to EcN strain, further confirming that the distinct band is a consequence of the cryptic plasmid curing (Additional file 1: Fig. S2). Based on the above results, we suggested that the expression of the Ag43c protein in EcNc is significantly higher compared to EcN, and this difference is attributed to the cryptic plasmids curing.

### Ag43 does not mediate cell self-aggregation

**Fig. 5:**
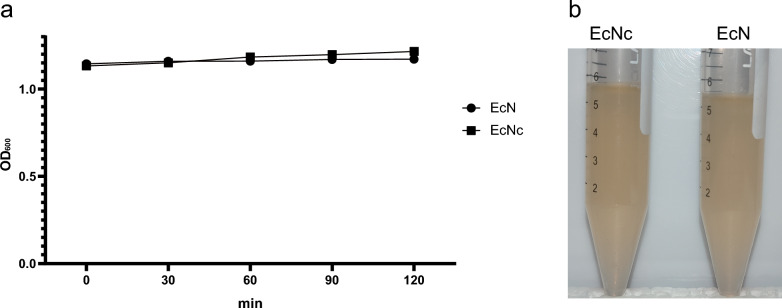
Aggregation test of strains. **a** Aggregation effect of EcN and EcNc strains (n = 3). **b** Self-sedimentation visualization of EcN and EcNc strains. The effects observed after a 2-h standing at room temperature for two bacterial strains with similar initial OD_600_ values

The above results have demonstrated a significant increase in Ag43 expression at the protein level following the cryptic plasmids curing. Studies have indicated that Ag43 can induce autoaggregation and sedimentation, thereby influencing bacterial colonization and infection (Henderson et al. 1997). Therefore, we conducted an autoaggregation assay to compare the self-aggregation abilities of EcN and EcNc. The results revealed that there was no significant change in the autoaggregation ability between EcNc and EcN (Fig. 5a), and no sedimentation was observed in these two strains (Fig. 5b).

## Discussion

Plasmids are extrachromosomal genetic elements that often encode features beneficial to the host organism, such as providing antibiotic resistance, heavy metal resistance, virulence factors, or metabolic functions, enhancing host adaptability (Al-Shayeb et al. 2022; Jacob and Hobbs 1974).

However, a substantial number of bacteria harbor ‘cryptic’ plasmids that lack evident beneficial functions (Fogarty et al. 2023). Certain cryptic plasmids may protect the carrier bacterium from attacks by bacteriophages (Feldgarden et al. 1995). EcN is one of the most commonly used chassis strains, harboring two cryptic plasmids with unknown phenotypes. The impact of cryptic plasmids curing in EcN requires further investigation.

In the present work, the impact of curing these two cryptic plasmids on EcN was explored. Firstly, the EcNc strain containing no cryptic plasmid was constructed by plasmid incompatibility. Comparison of the transcriptome data between EcN and EcNc revealed a small subset of differentially expressed genes, predominantly associated with amino acid metabolism. Intriguingly, massive increase in Ag43 expression caused by the cryptic plasmids curing in EcN strain. However, the curing of the cryptic plasmids selectively upregulates the expression of Ag43c, one of three distinct Ag43 variants. Previous studies on EcN have demonstrated that the expression of Ag43 is phase-variable, which is co-regulated by dam-methylase (positive regulation) and the cell redox sensor OxyR (negative regulation) (Henderson and Owen 1999). Despite these regulatory factors were found, the mechanism responsible for the specific upregulation of Ag43c following cryptic plasmids curing remains elusive.

Previous research indicates that Ag43 variants can mediate the self-aggregation of bacteria through the self-recognition ‘Velcro-handshake’ mechanism, which is associated with the oligomerization of the α-domain of Ag43 (Henderson et al. 1997; Heras et al. 2013). Aggregation experiments indicated that the Ag43c variant in EcN is incapable of mediating bacterial aggregation. Within uropathogenic *E. coli* strains (UPEC) CFT073, an *E. coli* strain highly homologous to EcN (van't Hof et al. 2022), there are two variants of the Ag43 that do not mediate bacterial aggregation in aggregation assays (Klemm et al. 2003). Another investigation suggested that under conditions of low protein concentration, the oligomerization of the α-domain of a Ag43 variant in the CFT073 strain does not occur, consequently failing to mediate the self-aggregation of bacteria (Heras et al. 2013). Another study revealed that the presence of fimbriation on bacteria eliminates Ag43-mediated self-aggregation but does not impact Ag43 expression (Hasman et al. 1999). F1C fimbriae are present in EcN (Kleta et al. 2014). While these findings suggest potential explanations for the increased expression of Ag43c in EcNc without a concomitant change in self-aggregation ability, further studies are needed.

Ag43 is regarded as having significant potential for displaying foreign proteins with large molecular weights and complex structures, surpassing other surface display systems (Jose 2006). Currently, Ag43 has been utilized for expressing exogenous protein fragments in numerous Gram-negative bacteria. For instance, the expression of the Ag43/Fcε3 chimeric protein on the surface of the *E. coli* strain Tan109 served as an effective asthma vaccine (Huang et al. 2014). Additionally, the Ag43 system has been employed to display β-glucosidase on the outer membrane of *E. coli*, facilitating the fermentation of crystalline cellulose during simultaneous saccharification and fermentation (SSF) (Muñoz-Gutiérrez et al. 2014). In another instance, red fluorescent protein (RFP) and cellulase (EC 3.2.1.4) were fused with Ag43, respectively, and can be displayed on the surface of *E. coli* (Jing et al. 2019). This implies that the EcNc strain, characterized by high expression of Ag43c, holds the potential to be used as a surface display strain.

### Supplementary Information

**Additional file 1: Figure S1.** Mass spectrometric analysis. (a-f) MS/MS spectra of peptides in Ag43a and Ag43b. **Figure S2.** Expression of Ag43 in different strains. Two recombinant cryptic plasmids expressing SOD were electrotransformed into EcNc, resulting in a strain designated as SEcNc. SDS-PAGE analysis was performed on whole-cell proteins and heat-extracted proteins of three strains—EcN, EcNc, and SEcNc.

**Additional file 2: Table S1.** Primers used for PCR in the study. **Table S2.** Primers used for qPCR in the study. **Table S3.** *Escherichia coli* strain SSGNw1 chromosome, complete genome. **Table S4.** Proteins identified by mass spectrometry. **Table S5.** Proteins identified by mass spectrometry (repeated).

## Data Availability

The raw data are available from the Sequence Read Archive with Bioproject accession PRJNA1045399.

## References

[CR1] Adolfsen KJ, Callihan I, Monahan CE, Greisen P, Spoonamore J, Momin M, Fitch LE, Castillo MJ, Weng L, Renaud L, Weile CJ, Konieczka JH, Mirabella T, Abin-Fuentes A, Lawrence AG, Isabella VM (2021). Improvement of a synthetic live bacterial therapeutic for phenylketonuria with biosensor-enabled enzyme engineering. Nat Commun.

[CR2] Ageorges V, Schiavone M, Jubelin G, Caccia N, Ruiz P, Chafsey I, Bailly X, Dague E, Leroy S, Paxman J, Heras B, Chaucheyras-Durand F, Rossiter AE, Henderson IR, Desvaux M (2019). Differential homotypic and heterotypic interactions of antigen 43 (Ag43) variants in autotransporter-mediated bacterial autoaggregation. Sci Rep.

[CR3] Ageorges V, Wawrzyniak I, Ruiz P, Bicep C, Zorgani MA, Paxman JJ, Heras B, Henderson IR, Leroy S, Bailly X, Sapountzis P, Peyretaillade E, Desvaux M (2023). Genome-wide analysis of antigen 43 (Ag43) variants: new insights in their diversity, distribution and prevalence in bacteria. Int J Mol Sci.

[CR4] Al-Shayeb B, Schoelmerich MC, West-Roberts J, Valentin-Alvarado LE, Sachdeva R, Mullen S, Crits-Christoph A, Wilkins MJ, Williams KH, Doudna JA, Banfield JF (2022). Borgs are giant genetic elements with potential to expand metabolic capacity. Nature.

[CR5] Avison MB, Walsh TR, Bennett PM (2001). pUB6060: a broad-host-range, DNA polymerase-I-independent ColE2-like plasmid. Plasmid.

[CR6] Benz I, Schmidt MA (2011). Structures and functions of autotransporter proteins in microbial pathogens. Int J Med Microbiol.

[CR7] Blum-Oehler G, Oswald S, Eiteljörge K, Sonnenborn U, Schulze J, Kruis W, Hacker J (2003). Development of strain-specific PCR reactions for the detection of the probiotic *Escherichia coli* strain Nissle 1917 in fecal samples. Res Microbiol.

[CR8] Dong M-M, Song L, Xu J-Q, Zhu L, Xiong L-B, Wei D-Z, Wang F-Q (2023). Improved cryptic plasmids in probiotic *Escherichia coli* Nissle 1917 for antibiotic-free pathway engineering. Appl Microbiol Biotechnol.

[CR9] Feldgarden M, Golden S, Wilson H, Riley MA (1995). Can phage defence maintain colicin plasmids in *Escherichia coli*?. Microbiology.

[CR10] Fogarty EC, Schechter MS, Lolans K, Sheahan ML, Veseli I, Moore R, Kiefl E, Moody T, Rice PA, Yu MK, Mimee M, Chang EB, McLellan SL, Willis AD, Comstock LE, Eren AM (2023). A highly conserved and globally prevalent cryptic plasmid is among the most numerous mobile genetic elements in the human gut. bioRxiv.

[CR11] Hasman H, Chakraborty T, Klemm P (1999). Antigen-43-mediated autoaggregation of *Escherichia coli* is blocked by fimbriation. J Bacteriol.

[CR12] Henderson IR, Owen P (1999). The major phase-variable outer membrane protein of *Escherichia coli* structurally resembles the immunoglobulin A1 protease class of exported protein and is regulated by a novel mechanism involving Dam and OxyR. J Bacteriol.

[CR13] Henderson IR, Meehan M, Owen P (1997). Antigen 43, a phase-variable bipartite outer membrane protein, determines colony morphology and autoaggregation in *Escherichia coli* K-12. FEMS Microbiol Lett.

[CR14] Henderson IR, Navarro-Garcia F, Desvaux M, Fernandez RC, Ala'Aldeen D (2004). Type V protein secretion pathway: the autotransporter story. Microbiol Mol Biol Rev.

[CR15] Heras B, Totsika M, Peters KM, Paxman JJ, Gee CL, Jarrott RJ, Perugini MA, Whitten AE, Schembri MA (2013). The antigen 43 structure reveals a molecular Velcro-like mechanism of autotransporter-mediated bacterial clumping. Proc Natl Acad Sci.

[CR16] Huang FY, Wang CC, Huang YH, Zhao HG, Guo JL, Zhou SL, Wang H, Lin YY, Tan G-H (2014). Ag43/Fcε3 chimeric protein expressed by a novel bacterial surface expression system as an effective asthma vaccine. Immunology.

[CR17] Jacob AE, Hobbs SJ (1974). Conjugal transfer of plasmid-borne multiple antibiotic resistance in *Streptococcus faecalis* var. *zymogenes*. J Bacteriol.

[CR18] Jing K, Guo Y, Ng IS (2019). Antigen-43-mediated surface display revealed in *Escherichia coli* by different fusion sites and proteins. Bioresour Bioprocess.

[CR19] Jose J (2006). Autodisplay: efficient bacterial surface display of recombinant proteins. Appl Microbiol Biotechnol.

[CR20] Kan A, Gelfat I, Emani S, Praveschotinunt P, Joshi NS (2020). Plasmid vectors for in vivo selection-free use with the probiotic *E. coli* Nissle 1917. ACS Synth Biol.

[CR21] Kim D, Paggi JM, Park C, Bennett C, Salzberg SL (2019). Graph-based genome alignment and genotyping with HISAT2 and HISAT-genotype. Nat Biotechnol.

[CR22] Klemm P, Hjerrild L, Gjermansen M, Schembri MA (2003). Structure-function analysis of the self-recognizing Antigen 43 autotransporter protein from *Escherichia coli*. Mol Microbiol.

[CR23] Kleta S, Nordhoff M, Tedin K, Wieler LH, Kolenda R, Oswald S, Oelschlaeger TA, Bleiss W, Schierack P (2014). Role of F1C fimbriae, flagella, and secreted bacterial components in the inhibitory effect of probiotic *Escherichia coli* Nissle 1917 on atypical enteropathogenic *E. coli* infection. Infect Immun.

[CR24] Koh DW-S, Tay J-H, Gan SK-E (2022). Engineering Ag43 signal peptides with bacterial display and selection. Methods Protocols.

[CR25] Liu X, Wang D, Wang H, Feng E, Zhu L, Wang H (2012). Curing of plasmid pXO1 from *Bacillus* anthracis using plasmid incompatibility. PLoS ONE.

[CR26] Love MI, Huber W, Anders S (2014). Moderated estimation of fold change and dispersion for RNA-seq data with DESeq2. Genome Biol.

[CR27] Muñoz-Gutiérrez I, Moss-Acosta C, Trujillo-Martinez B, Gosset G, Martinez A (2014). Ag43-mediated display of a thermostable β-glucosidase in *Escherichia coli* and its use for simultaneous saccharification and fermentation at high temperatures. Microb Cell Fact.

[CR28] Owen P, Meehan M, de Loughry-Doherty H, Henderson I (1996). Phase-variable outer membrane proteins in *Escherichia coli*. FEMS Immunol Med Microbiol.

[CR29] Praveschotinunt P, Duraj-Thatte AM, Gelfat I, Bahl F, Chou DB, Joshi NS (2019). Engineered *E. coli* Nissle 1917 for the delivery of matrix-tethered therapeutic domains to the gut. Nat Commun.

[CR30] Putri GH, Anders S, Pyl PT, Pimanda JE, Zanini F (2022). Analysing high-throughput sequencing data in Python with HTSeq 2.0. Bioinformatics.

[CR31] Remer KA, Bartrow M, Roeger B, Moll H, Sonnenborn U, Oelschlaeger TA (2009). Split immune response after oral vaccination of mice with recombinant *Escherichia coli* Nissle 1917 expressing fimbrial adhesin K88. Int J Med Microbiol.

[CR32] Roche A, McFadden J, Owen P (2001). Antigen 43, the major phase-variable protein of the *Escherichia coli* outer membrane, can exist as a family of proteins encoded by multiple alleles. Microbiology.

[CR33] Sarnelli G, Del Re A, Pesce M, Lu J, Esposito G, Sanseverino W, Corpetti C, BasiliFranzin S, Seguella L, Palenca I, Rurgo S, De Palma FDE, Zilli A, Esposito G (2023). Oral immunization with *Escherichia coli* Nissle 1917 expressing SARS-CoV-2 spike protein induces mucosal and systemic antibody responses in mice. Biomolecules.

[CR34] Seo E-J, Weibel S, Wehkamp J, Oelschlaeger TA (2012). Construction of recombinant *E. coli* Nissle 1917 (EcN) strains for the expression and secretion of defensins. Int J Med Microbiol.

[CR35] Sonnenborn U, Schulze J (2009). The non-pathogenic *Escherichia coli* strain Nissle 1917—features of a versatile probiotic. Microb Ecol Health Dis.

[CR36] Teng G, Liu Z, Liu Y, Wu T, Dai Y, Wang H, Wang W (2022). Probiotic *Escherichia coli* Nissle 1917 expressing Elafin protects against inflammation and restores the gut microbiota. Front Microbiol.

[CR37] van't Hof M, Mohite OS, Monk JM, Weber T, Palsson BO, Sommer MOA (2022). High-quality genome-scale metabolic network reconstruction of probiotic bacterium *Escherichia coli* Nissle 1917. BMC Bioinform.

[CR38] Wu T, Hu E, Xu S, Chen M, Guo P, Dai Z, Feng T, Zhou L, Tang W, Zhan L, Fu X, Liu S, Bo X, Yu G (2021). clusterProfiler 4.0: a universal enrichment tool for interpreting omics data. Innovation.

[CR39] Ying Y, Bing-ming O, Jun Z, Yi Y, Qian Z, Wei-feng Y, Peng-peng X, Guo-qiang Z (2016) Establishment of the method for cryptic plasmid curing in *Escherichia coli* Nissle 1917. Chin J Vet Sci 36(06):933–937+943. 10.16303/j.cnki.1005-4545.2016.06.09

[CR40] Zainuddin HS, Bai Y, Mansell TJ (2019). CRISPR-based curing and analysis of metabolic burden of cryptic plasmids in *Escherichia coli* Nissle 1917. Eng Life Sci.

[CR41] Zhou J, Li M, Chen Q, Li X, Chen L, Dong Z, Zhu W, Yang Y, Liu Z, Chen Q (2022). Programmable probiotics modulate inflammation and gut microbiota for inflammatory bowel disease treatment after effective oral delivery. Nat Commun.

