# Supplementary material for: Unveiling the impact of cryptic plasmids curing on Escherichia coli Nissle 1917: massive increase in Ag43c expression
Source: AMB Express. 2024 Apr 28;14:48. doi: 10.1186/s13568-024-01681-9 (PMC11056357; doi:10.1186/s13568-024-01681-9)
Supplement: Supplementary file 1 — Additional file 1: Figure S1. Mass spectrometric analysis. (a-f) MS/MS spectra of peptides in Ag43a and Ag43b. Figure S2. Expression of Ag43 in different strains. Two recombinant cryptic plasmids expressing SOD were electrotransformed into EcNc, resulting in a strain designated as SEcNc. SDS-PAGE analysis was performed on whole-cell proteins and heat-extracted proteins of three strains—EcN, EcNc, and SEcNc. [file 13568_2024_1681_MOESM1_ESM.docx]

## Additional Figures

Journal name: AMB Express

**Manuscript Title:** Unveiling the Impact of Cryptic Plasmids Curing on *Escherichia coli* Nissle 1917: Massive increase in Ag43c expression

**The names of the authors:** Qi Lin^1,2^, Zhuo Jiang^1^, Bo Zhong^1^, Jian-qing Chen^1,2^, Zheng-bing lv^1,2^, Zuo-ming Nie^1,2*^

1 College of Life Sciences and Medicine, Zhejiang Sci-Tech University, Hangzhou 310018, China

2 Centre for Bioreactor and Protein Drug Research, Shaoxing Biomedical Research Institute of Zhejiang Sci-Tech University Co., Ltd, Shaoxing 312075, China

*To whom correspondence should be addressed. Tel: +8613958160384; Email: [nzm16@tsinghua.org.cn](mailto:nzm16@tsinghua.org.cn).


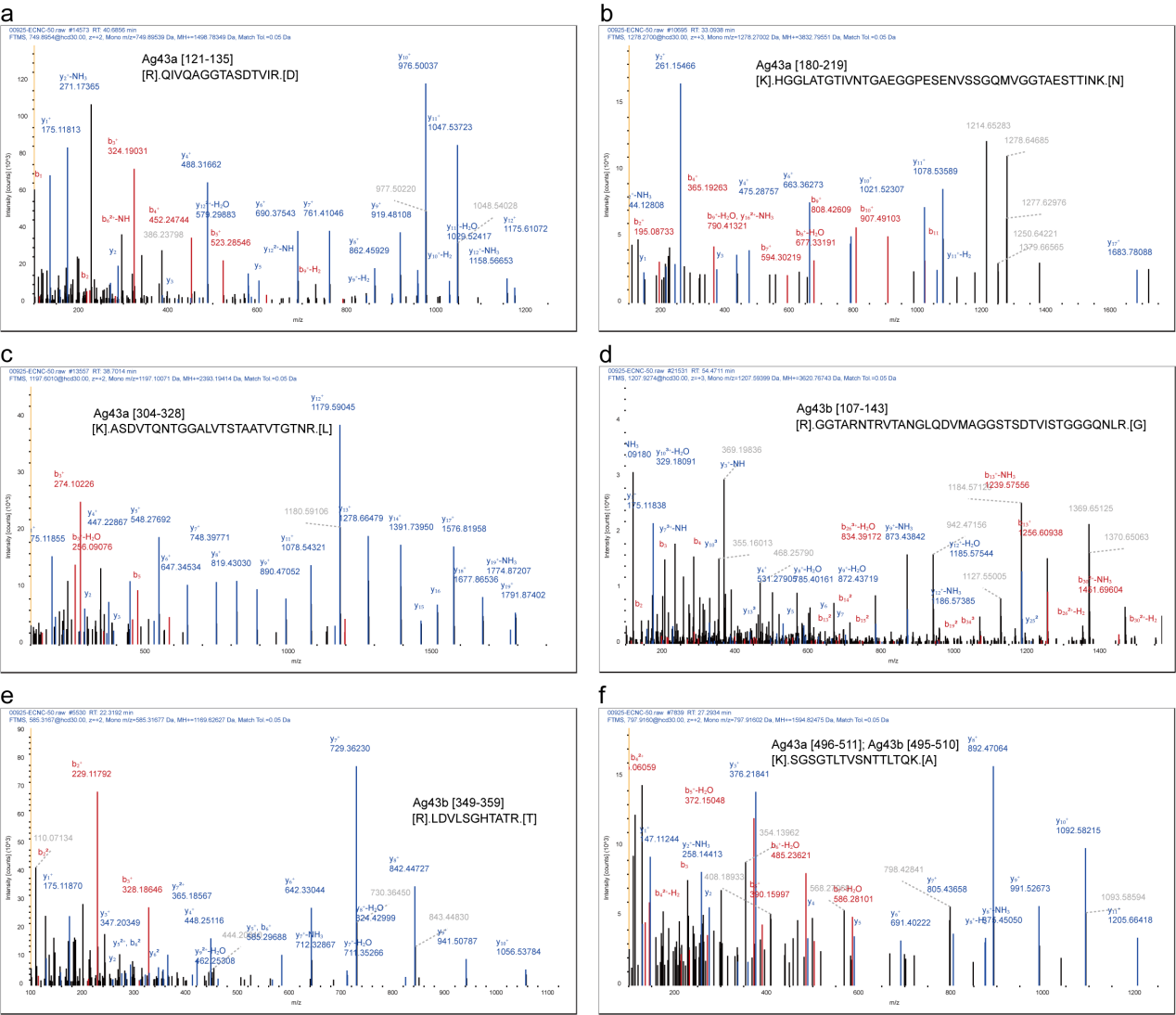


**Figure S1 Mass spectrometric analysis**

(a-f) MS/MS spectra of peptides in Ag43a and Ag43b.


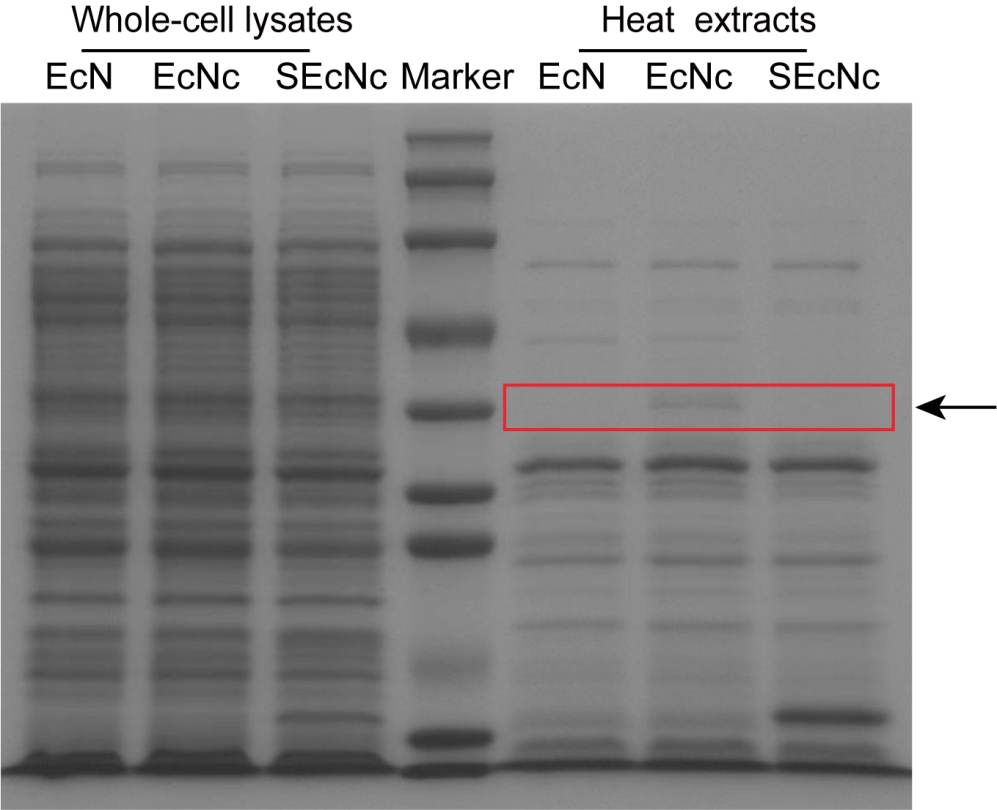


**Figure S2 Expression of Ag43 in different strains**

Two recombinant cryptic plasmids expressing SOD were electrotransformed into EcNc, resulting in a strain designated as SEcNc. SDS-PAGE analysis was performed on whole-cell proteins and heat-extracted proteins of three strains—EcN, EcNc, and SEcNc.
